# Loss of Myostatin Alters Mitochondrial Oxidative Phosphorylation, TCA Cycle Activity, and ATP Production in Skeletal Muscle

**DOI:** 10.3390/ijms232415707

**Published:** 2022-12-11

**Authors:** Xueqiao Wang, Zhuying Wei, Mingjuan Gu, Lin Zhu, Chao Hai, Anqi Di, Di Wu, Chunling Bai, Guanghua Su, Xuefei Liu, Lei Yang, Guangpeng Li

**Affiliations:** 1 State Key Laboratory of Reproductive Regulation and Breeding of Grassland Livestock, College of Life Science, Inner Mongolia University, Hohhot 010070, China; 2 College of Animal Science, Inner Mongolia Agricultural University, Hohhot 010018, China

**Keywords:** myostatin, mitochondria, energy metabolism, oxidative phosphorylation, TCA cycle, ATP

## Abstract

**Simple Summary:**

Myostatin (MSTN) negatively regulates skeletal muscle growth. Although the role of MSTN in muscle hypertrophy can be investigated in depth, studies of MSTN in mitochondrial energy metabolism in muscle would be valuable. In this study, we evaluated the importance of MSTN in regulating mitochondrial energy metabolism in MSTN-knockout (*Mstn^−/−^*) mice and explored the possible mechanisms. A loss of MSTN inhibits oxidative phosphorylation, alters TCA cycle activity, and impairs ATP production in skeletal muscles. These changes may be achieved through TGF-β-Smad2/3. These results suggest that MSTN may be an important regulator of mitochondrial energy homeostasis in mice.

**Abstract:**

Myostatin (MSTN) is an important negative regulator of skeletal muscle growth in animals. A lack of MSTN promotes lipolysis and glucose metabolism but inhibits oxidative phosphorylation (OXPHOS). Here, we aimed to investigate the possible mechanism of MSTN regulating the mitochondrial energy homeostasis of skeletal muscle. To this end, MSTN knockout mice were generated by the CRISPR/Cas9 technique. Expectedly, the MSTN null (*Mstn*^−/−^) mouse has a hypermuscular phenotype. The muscle metabolism of the *Mstn^−/−^* mice was detected by an enzyme-linked immunosorbent assay, indirect calorimetry, ChIP-qPCR, and RT-qPCR. The resting metabolic rate and body temperature of the *Mstn^−/−^* mice were significantly reduced. The loss of MSTN not only significantly inhibited the production of ATP by OXPHOS and decreased the activity of respiratory chain complexes, but also inhibited key rate-limiting enzymes related to the TCA cycle and significantly reduced the ratio of NADH/NAD+ in the *Mstn^−/−^* mice, which then greatly reduced the total amount of ATP. Further ChIP-qPCR results confirmed that the lack of MSTN inhibited both the TCA cycle and OXPHOS, resulting in decreased ATP production. The reason may be that Smad2/3 is not sufficiently bound to the promoter region of the rate-limiting enzymes Idh2 and Idh3a of the TCA cycle, thus affecting their transcription.

## 1. Introduction

Myostatin (MSTN) is a well-known myokine, and a large number of studies in the past decades have shown that natural or artificial mutations in MSTN are associated with double-muscle phenotypes in cattle, sheep, goat, horse, pig, rabbit, chicken, and other domestic animals [1]. Mutation of MSTN or treatment with myostatin antibody/inhibitors such as YN41 [2], FST288-Fc [3], muSRK-015P [4], LTBP4 [5], and myostatin propeptide (a natural inhibitor of mature myostatin) [6] can effectively increase muscle mass and counteract muscle atrophy [7]. An MSTN deletion results in larger muscle mass but not greater muscle fiber strength [8], leading to an impairment in muscle power generation capacity [9] and a significant decrease in specific maximal strength [10]. The muscles of the MSTN null (*Mstn^−/−^*) mice were weaker than those of the wild-type mice [11].

Muscle strength and force are related to the muscle fiber type and ATP energy supply. Skeletal muscle is composed of slow (Type I) and fast (Type II) fibers, which convert chemical energy into energy for contraction and metabolism [12]. Slow fibers are richer in mitochondria and rely on oxidative phosphorylation (OXPHOS) as the preferred metabolic route to produce ATP. Fast fibers, on the other hand, have fewer mitochondria and prefer glycolysis to produce ATP [13]. A larger proportion of glycolytic fibers and a reduced proportion of oxidative fibers occur in the extensor digitorum longus (EDL) of the *Mstn* knockouts [14]. Consistently, MSTN mutation leads to reduced ATP synthesis [15]. The mitochondria play a critical role in providing ATP through the OXPHOS process. A loss of MSTN expression is associated with a decrease in mitochondrial content [11] and mitochondrial respiration [16]. MSTN mutations also reduce mitochondrial respiratory chain complex activity and OXPHOS capacity [17,18]. The genes related to the mitochondrial respiratory chains for ATP synthesis were significantly down-regulated in MSTN-deficient mice [19]. MSTN regulated the G protein-coupled receptor (GPCR) kinase 2 (GRK2) on mitochondrial respiration, but the overexpression of GRK2 did not prevent MSTN-mediated mitochondrial respiratory damage, suggesting that GRK2 is not the main factor between MSTN and mitochondrial respiration [20]. Moreover, the effects of MSTN deficiency on mitochondrial function in different tissues are different. In adipocytes, the inactivation of MSTN increased mitochondrial biogenesis [21]. The reason why MSTN affects energy metabolism related to mitochondrial respiration needs further investigation.

MSTN is a member of the transforming growth factor-β (TGF-β) superfamily, and deletion of MSTN affects gene expression, often through the TGF-β signaling pathway. Thus, the downstream signaling molecules, the SMAD family members, are unable to enter the nucleus to function as transcription factors [22]. In skeletal muscles, MSTN binds to type I (ALK4/5) and type II (ActRIIA/B) receptors, initiating the phosphoinositide 3-kinase (PI3K) or Smad2/3 signal pathway and regulating the balance between anabolism and catabolism [23]. An MSTN deficiency can inhibit the TGF-β signaling pathway, down-regulate the expression of Smad2/3, and promote the expression of AMP kinase to regulate glucose metabolism in bovine skeletal muscle [24]. Similarly, Smad2/3 expression was inhibited in MSTN-deficient bovine myocardium to regulate the activation of glycolysis-related enzymes [25].

The TCA cycle plays an important role in the energy homeostasis of skeletal muscle, and its metabolites can induce the transformation of muscle fibers [26]. As an important rate-limiting enzyme in the TCA cycle, isocitrate dehydrogenase (IDH) is involved in the maintenance of muscle energy homeostasis [27]. As we know, an MSTN knockout can change the type of muscle fibers [28,29]. However, the relationship between MSTN and IDH in skeletal muscle energy metabolism remains unclear.

Therefore, MSTN mutation can lead to increased muscle mass and disrupted energy metabolism. However, the mechanism of muscle energy change in MSTN-mutated animals remains unclear. In this study, we investigated the effect of MSTN on muscle energy metabolism. Using the CRISPR/Cas9 technique to generate *Mstn^−/−^* mice, we found that skeletal muscle ATP accumulation was significantly reduced in the *Mstn^−/−^* mice compared to the wild-type controls. To further understand the ATP changes, muscle OXPHOS and glycolysis analyses were performed. Although the glycolysis was enhanced, the OXPHOS in the *Mstn^−/−^* muscles was significantly lower than that in the wild-type muscles. Combined with the mitochondrial respiratory chain complex and TCA cycle enzyme activities, we hypothesized that the MSTN deficiency decreased the expression of Smad 2/3 downstream of the TGF-β signaling pathway, which prevented it from acting as a transcription factor, inhibited the expression of TCA-cycle related rate-limiting enzymes and thus inhibited the efficiency of the TCA cycle, resulting in decreased ATP synthesis. This leads to a decrease in mitochondrial respiratory function.

## 2. Results

### 2.1. Mstn^−/−^ Mice Developed a Typical Muscle Hypertrophy Phenotype

**Figure 1:**
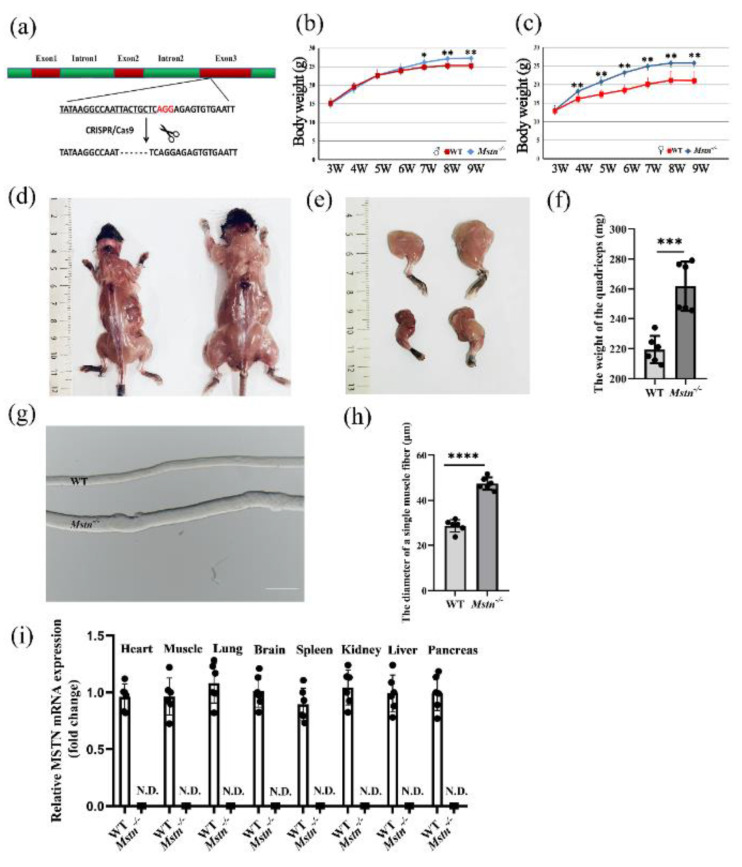
Comparison of muscle morphology and Myostatin (MSTN) expression between wild-type (WT) and MSTN null (*Mstn^−/−^)* mice. (**a**) Schematic diagram of the gene mutation site. (**b**) Growth-rate comparison for male mice. (**c**) Growth-rate comparison for female mice. (**d**) Morphological appearance of WT (left) and *Mstn^−/−^* (right) mice muscles. Scale bars, 1 cm. (**e**) Morphological appearance of forelimbs (bottom) and hindlimbs (top) in WT (left) and *Mstn^−/−^* (right) mice. Scale bars, 1 cm. (**f**) Weight for the quadriceps. Each dot presents the quadriceps weight of each mouse. (**g**) Muscle fibers of the quadriceps in WT (top) and *Mstn^−/−^* (bottom) mice. Scale bars, 100 μm. (**h**) Quantitative analysis of muscle fiber diameter. Each dot presents the muscle fiber diameter of each mouse. (**i**) Real-time PCR analyses of *Mstn* expression in different organs. N.D., not detectable. All the data are presented as mean ± SD. Compared with the control group, * *p* < 0.05, ** *p* < 0.01, *** *p* < 0.001, **** *p* < 0.0001; Student’s *t*-tests were used to calculate the *p*-values. We used n = 6 mice per group. Except where noted, each dot represents a mouse.

We generated an *Mstn^−/−^* mouse model by a unique method based on CRISPR/Cas9, with a homozygous 6-bp deletion in the third exon nt175-180 (Figure 1a). The *Mstn^−/−^* mice were significantly heavier than the controls from 4 weeks old (females) and 7 weeks old (males). This weight remained stable after 8 weeks old (Figure 1b,c). The muscles of the *Mstn^−/−^* mice were larger than those of the wild-type mice (8 weeks of age, male, Figure 1d,e), and the muscle weights were higher in all regions than in the wild-type mice (Supplementary Table S1). The most significant difference was in the quadriceps (8 weeks old, male, Figure 1f, *p* < 0.001), and their muscle fibers were coarser than those of the wild type (Figure 1g,h). In addition, the heart weight, kidney weight, and liver weight of the *Mstn^−/−^* mice were also significantly greater than those of the control mice (Supplementary Table S2). Moreover, the mRNA expression of *Mstn* decreased significantly in the organs and tissues of the *Mstn^−/−^* mice (Figure 1i and Figure S1a). Together, these results proved the successful generation of the *Mstn^−/−^* mice.

### 2.2. Mstn^−/−^ Mice Significantly Reduced Muscle ATP Level

**Figure 2:**
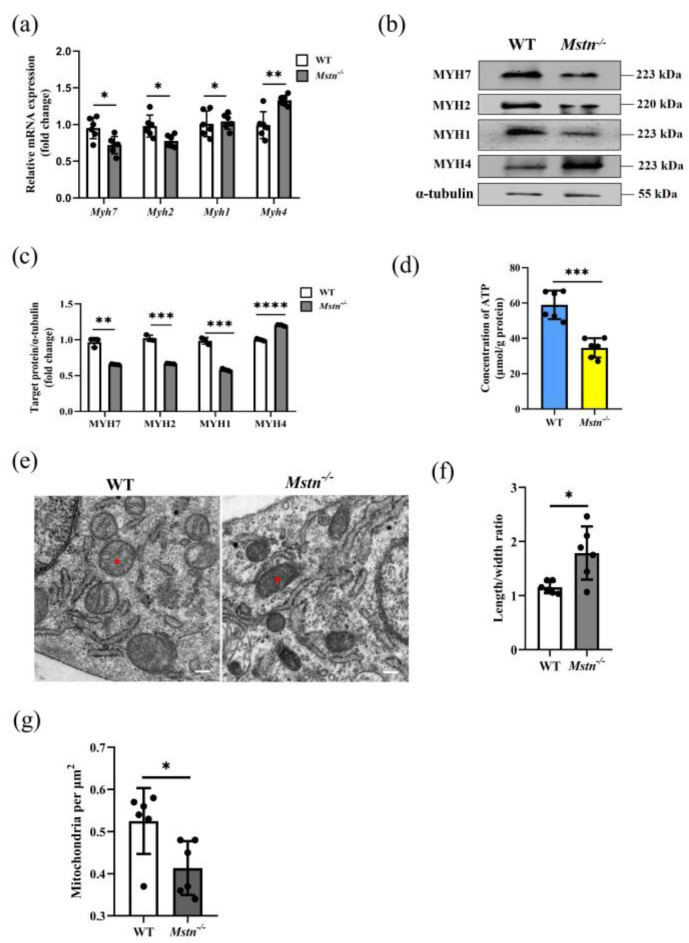
The type of muscle energy metabolism is changed in *Mstn^−/−^* mice. (**a**) Expression of mRNA for myosin heavy chain in quadriceps. (**b**) Expression of protein for myosin heavy chain in quadriceps. (**c**) The quadriceps proteins of 6 WT mice and *Mstn^−/−^* mice were collected and analyzed quantitatively in triplicate Each dot presents a repeat. (**d**) The total ATP in the WT and *Mstn^−/−^* mice quadriceps. (**e**) Transmission electron microscopy (TEM) of muscle cell mitochondria from WT (left) and *Mstn^−/−^* mice (Right). The red stars present the location of the mitochondria. Scale bars, 100 nm. (**f**) Quantification of TEM: the ratio of mitochondrial length to width from (**e**). (**g**) Quantification of TEM mitochondria: the number of mitochondria per μm^2^ from (**e**). Data presented as mean ± SD. Compared with the control group, * *p* < 0.05, ** *p* < 0.01, *** *p* < 0.001, **** *p* < 0.0001; Student’s *t*-tests were used to calculate the *p*-values. We used n = 6 mice per group. Except where noted, each dot represents a mouse.

Different types of muscle fibers in the muscle metabolize differently, determining the muscle’s capacity for strength and endurance. The quadriceps are adapted to produce the greatest absolute force [30]. We used molecular markers of muscle fiber types to infer the changes in muscle fibers. The mRNA and protein expression of myosin heavy chain in the quadriceps were detected. The expression of the molecular markers MYH7, MYH2, and MYH1 of type Ⅰ, type Ⅱa, and type Ⅱx muscle fibers were significantly decreased in the *Mstn^−/−^* mice. There was an increase in the expression of the molecular markers of fibers Ⅱb in the *Mstn^−/−^* mice, likely reflecting the presence of more type Ⅱb fibers (Figure 2a–c). Since the type and composition of muscle fibers are changed, the energy supply of muscle may change, so we measured the total ATP content in the muscle. The total ATP concentration in the *Mstn^−/−^* mice was significantly reduced compared to the WT mice. (Figure 2d and Figure S2a. In Figure 2a, WT vs. *Mstn^−/−^*: 58.95 ± 8.05 vs. 34.64 ± 5.47 μmol/g protein, *p* = 0.0001,). Compared with the wild-type mice, the muscle cells of the *Mstn^−/−^* mice were observed by transmission electron microscopy (TEM) with thinner and smaller mitochondria, and the number of mitochondria was significantly lower than that of the wild-type mice (Figure 2e–g).

### 2.3. Reduced ATP Level of Mstn^−/−^ Mice Resulted from Decreased OXPHOS Activity

**Figure 3:**
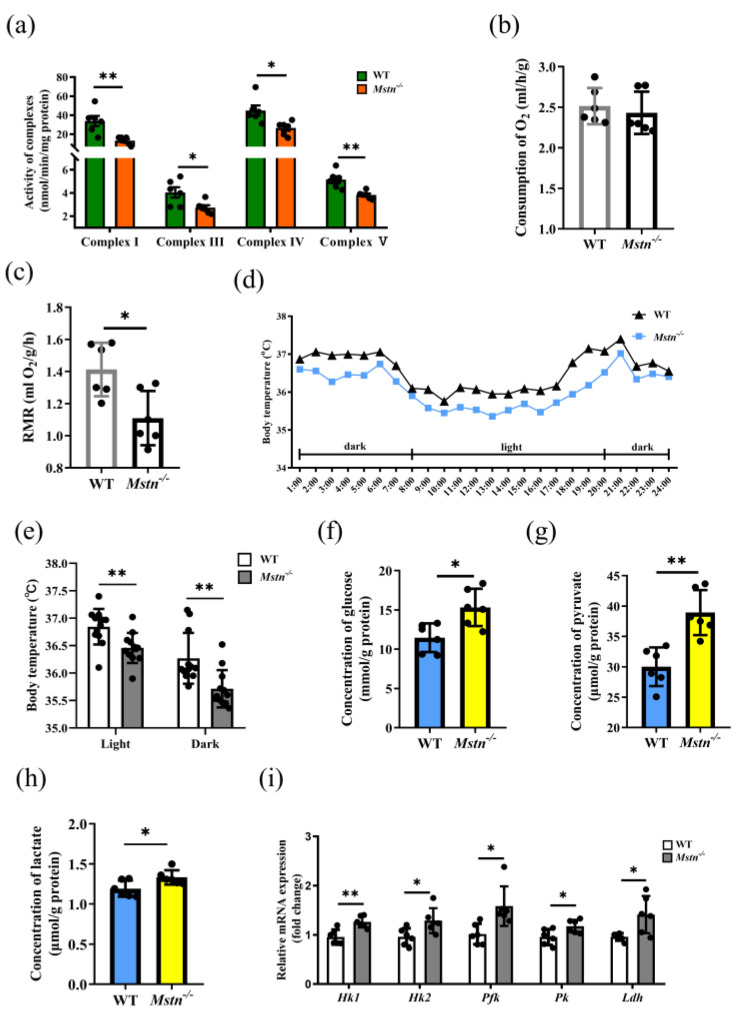
Muscular OXPHOS was inhibited in *Mstn^−/−^* mice. (**a**) Mitochondrial complex activity in quadriceps of WT and *Mstn^−/−^* mice. (**b**) The total O_2_ uptake levels. (**c**) RMR of WT and *Mstn^−/−^* mice. (**d**) The body temperature of WT and *Mstn^−/−^* mice. (**e**) The light and dark body temperature of WT and *Mstn^−/−^* mice. Each dot represents the average temperature of the mice at one time point. (**f**) The glucose concentrations in quadriceps of WT and *Mstn^−/−^* mice. (**g**) The pyruvate concentrations in quadriceps of WT and *Mstn^−/−^* mice. (**h**) The lactate concentrations in quadriceps of WT and *Mstn^−/−^* mice. (**i**) Expression of mRNA for key rate-limiting enzymes involved in glycolysis in quadriceps. All data except (**d**) are presented as mean ± SD. Compared with the control group, * *p* < 0.05, ** *p* < 0.01; Student’s *t*-tests were used to calculate the *p*-values. We used n = 6 mice per group. Except where noted, each dot represents a mouse.

The decrease in ATP in the muscles parallels the changes in OXPHOS and glycolysis in the muscle fibers. To determine whether the ATP reduction results from the OXPHOS or the glycolysis, we investigated the OXPHOS rates in situ by examining the activities of mitochondrial respiration complexes Ⅰ, Ⅲ, Ⅳ, and Ⅴ, and the glycolysis rates by the product quantity and enzyme expression. In comparison with the control group, the activities of complexes Ⅰ, Ⅲ, Ⅳ, and Ⅴ in the *Mstn^−/−^* muscles were all significantly decreased (Figure 3a and Figure S2b. In Figure 3a, WT vs. *Mstn^−/−^*: complex Ⅰ, 33.90 ± 12.88 vs. 13.39 ± 4.01 nmol/min/mg protein, *p* = 0.004; complex Ⅲ, 4.05 ± 1.09 vs. 2.73 ± 0.53 nmol/min/mg protein, *p* = 0.024; complex Ⅳ, 44.89 ± 13.05 vs. 26.56 ± 6.70 nmol/min/mg protein, *p* = 0.013; and complex Ⅴ, 5.14 ± 0.73 vs. 3.82 ± 0.33 nmol/min/mg protein, *p* = 0.003). The indirect calorimetry to measure the oxygen consumption and energy expenditure showed no change in the total oxygen consumption compared to the wild type (Figure 3b), but a significant reduction in the resting metabolic rate (RMR, WT vs. *Mstn^−/−^*: 1.40 ± 0.120 mL O_2_/g/h vs. 1.11 ± 0.116 mL O_2_/g/h, *p* = 0.015, Figure 3c). The body temperatures detected by continuous measurements indicated that the *Mstn^−/−^* mice were lower than the controls at each detected time point (Figure 3d,e). As a result, the *Mstn^−/−^* mice had a lower RMR and body temperature, consistent with reduced mitochondrial complex activity.

Glucose pyruvate and lactate were significantly increased in the muscles of the *Mstn^−/−^* mice (Figure 3f–h and Figure S2b–d). Meanwhile, the glycolytic genes Hexokinase 1 (*Hk1)*, Hexokinase 2 (*Hk2*), 6-phosphofructokinase (*Pfk1*), Pyruvate kinase (*Pk*), and Lactic dehydrogenase (*Ldh)* were significantly up-regulated (Figure 3i and Figure S1b). These results suggested that OXPHOS was inhibited while glycolysis was promoted in the muscles of the *Mstn^−/−^* mice.

### 2.4. The TCA Cycle Does Not Provide Sufficient Substrates for OXPHOS in Mstn^−/−^ Muscles

**Figure 4:**
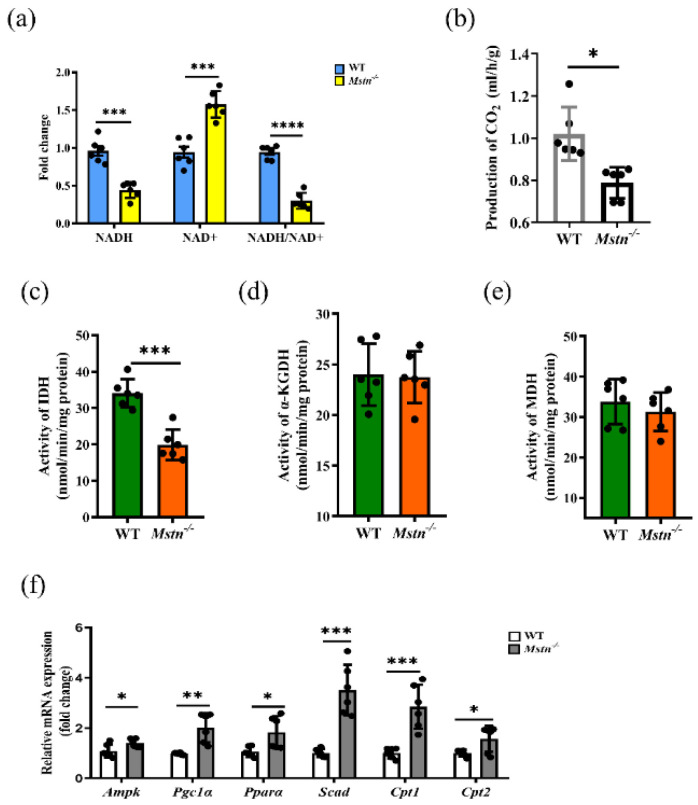
*Mstn* gene knockout-inhibited OXPHOS via the TCA cycle. (**a**) Concentrations of NADH and NAD+ in quadriceps of WT and *Mstn^−/−^* mice. (**b**) The production of CO_2_ in WT and *Mstn^−/−^* mice breath. (**c**) IDH activities in quadriceps of WT and *Mstn^−/−^* mice. (**d**) α-KGDH activities in quadriceps of WT and *Mstn^−/−^* mice. (**e**) MDH activities in quadriceps of WT and *Mstn^−/−^* mice. (**f**) Expression of mRNA for the gene involved in β-oxidation in quadriceps. All the data are presented as mean ± SD. Compared with the control group, * *p* < 0.05, ** *p* < 0.01, *** *p* < 0.001, **** *p* < 0.0001; Student’s *t*-tests were used to calculate the *p*-values. We used n = 6 mice per group. Each dot presents a mouse.

Complex I is the rate-limiting step of the electron transport chain [31]. As the substrate of complex I, the content of NADH limits the complex I activity. Compared to the wild-type control, the NADH/NAD+ ratio in the *Mstn^−/−^* muscles was significantly decreased, with the NADH level decreasing by about 52%, and the NAD+ increasing by about 63% (Figure 4a and Figure S2e). The mitochondrial NADH concentration is mainly attributed to the TCA cycle and fatty acid oxidation. The TCA cycle rate of the *Mstn^−/−^* muscles was down-regulated, in parallel to the decrease in CO_2_ exhalation (WT vs. *Mstn^−/−^*: 1.06 ± 0.07 mL/h/g vs. 0.80 ± 0.03 mL/h/g, *p* = 0.016, Figure 4b). Compared with the WT group, the IDH complex activity in the *Mstn^−/−^* muscles was significantly decreased (Figure 4c and Figure S2f). In Figure 4c, WT vs. *Mstn^−/−^*: 34.03 ± 3.88 vs. 19.91 ± 4.18 nmol/min/mg protein, *p* = 0.0001), while α-ketoglutarate dehydrogenase (α-KGDH) and Malate dehydrogenase (MDH) showed no significant difference (Figure 4d,e and Figure S2g,h). Adenosine monophosphate-activated protein kinase (AMPK), Peroxisome proliferator-activated receptor gamma coactivator 1 alpha (PGC-1α), and Peroxisome proliferator-activated receptor-alpha (PPARα), key members of the fatty acid β-oxidation pathway AMPK-PPAR, in the *Mstn^−/−^* muscles had significantly higher expressions than those in the controls (Figure 4f and Figure S1c). In the *Mstn^−/−^* mice, the expressions of Short-chain acyl-CoA dehydrogenase (Scad), Carnitine palmitoyltransferase 1 (Cpt1), and Carnitine palmitoyltransferase 2 (Cpt2), which catalyze fatty acid β-oxidation, were also up-regulated (Figure 4f). These results indicate that the reduction of NADH is mainly attributed to the TCA cycle rather than the fatty acid β-oxidation, and the downregulation of the IDH activity of the key rate-limiting enzyme is the main reason for the decrease in the TCA cycle activity.

### 2.5. Loss of MSTN Disrupted Promotive Regulation of Idh2 and Idh3a through Smad2/3

**Figure 5:**
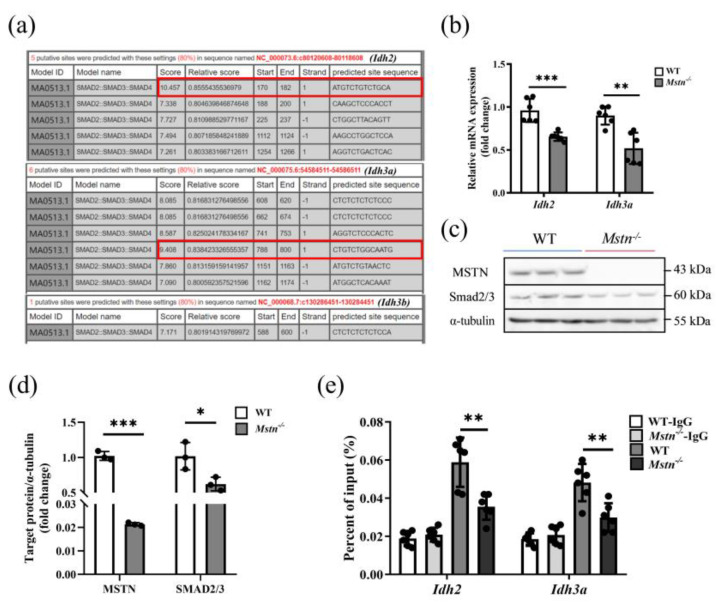
MSTN regulated the TCA cycle via Smad2/3. (**a**) Analysis of Smad2/3 binding sites in *Idh2*, *Idh3a*, and *Idh3b* promotor sequences. The red box line is the binding site selected in this study. (**b**) Expression of *Idh2* and *Idh3a* mRNA in the quadriceps of WT and *Mstn^−/−^* mice. (**c**) The proteins of 6 WT and *Mstn^−/−^* mice were collected and triplicated in three lanes to show the protein expression of Smad2/3 and MSTN. (**d**) The protein expression of Smad2/3 and MSTN in (**c**) was quantitatively analyzed. Each dot presents a repeat. (**e**) ChIP-qPCR detected the binding of Smad2/3 to the *Idh2* and *Idh3a* promoter regions in *Mstn^−/−^* mice quadriceps. (**b**,**d**,**e**) The data are presented as mean ± SD. Compared with the control group, * *p* < 0.05, ** *p* < 0.01, *** *p* < 0.001; Student’s *t*-tests were used to calculate the *p*-values. We used n = 6 mice per group. Except where noted, each dot represents a mouse.

The activity of IDH, a key rate-limiting enzyme in the TCA cycle, was significantly inhibited. We explored whether the expression of the IDH gene was changed. Next, we attempted to figure out the molecular relationship between MSTN and the *Idh1*, *Idh2*, *Idh3a*, and *Idh3b* genes in muscle, which are expressed and function in skeletal muscle in the form of IDH2, IDH3A, and IDH3B in the mitochondria. We hypothesized that MSTN might act as the transcriptional regulator via the TGF-β signaling pathway. We analyzed the promoter sequences of *Idh2*, *Idh3a,* and *Idh3b* and found that only the promoter sequences of *Idh2* and *Idh3a* had putative binding sites for Smad2/3 (Figure 5a). Compared with the WT mice, the mRNA expression levels of *Idh2* and *Idh3a* in the *Mstn*^−/−^ muscles were significantly reduced (Figure 5b and Figure S1d). The MSTN and Smad2/3 proteins were significantly decreased in the *Mstn*^−/−^ muscle (Figure 5c,d). Using a Chromatin immunoprecipitation (ChIP)-qPCR assay, we found that the Smad2/3 antibody could bind to *Idh2* and *Idh3a* promoters (Figure 5e). These results suggest that MSTN can regulate the transcription of *Idh2* and *Idh3a* through Smad2/3, and the deletion of MSTN reduces the expression and enzyme activity of the *Idh2* and *Idh3a* genes.

## 3. Discussion

### 3.1. The Effect of Mstn^−/−^ on Muscle Morphology

**Figure 6:**
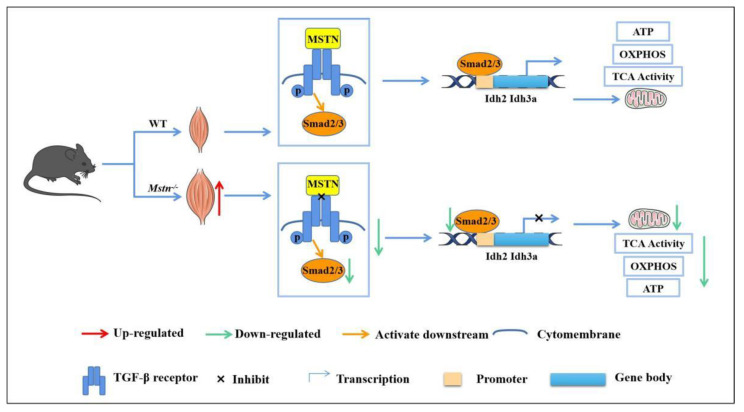
MSTN regulates the expression of IDH through Smad2/3 to affect mitochondrial energy metabolism. Upon MSTN knockdown, binding of MSTN to TGF-β receptors is reduced. This inhibits the entry of Smad2/3 into the nucleus to function as a transcription factor. Less Smad2/3 binds to the promoter regions of *Idh2* and *Idh3a*, and the gene is unable to initiate transcription. The TCA cycle is inhibited, altering mitochondrial morphology and mitochondrial function, and inhibiting ATP production.

In this study, we identified a novel pathway by which MSTN regulates energy metabolism (Figure 6). Consistent with other reports, we found that an MSTN loss resulted in an increase in muscle mass [32,33]. In our study, the muscle mass in different regions of mice increased, among which the quadriceps muscle mass of *Mstn^−/−^* male 8-week-old mice had the biggest difference from the wild type. It is well known that the knockout of MSTN increases the relative proportion of glycolytic fibers (type Ⅱb) at the expense of the hindlimb muscles of oxidative (type Ⅰ) [34]. We used molecular markers of muscle fiber types to infer the changes in muscle fibers. The expression of molecular markers MYH7, MYH2, and MYH1 of type Ⅰ, type Ⅱa, and type Ⅱx muscle fibers was significantly decreased. There was an increase in the expression of the molecular markers of fibers Ⅱb, likely reflecting the presence of more fibers of type Ⅱb. This is consistent with the results of the *Mstn^−/−^* mice EDL, but different from the results of the soleus [14]. This variation may be due to the different muscle types.

The previous study showed that inhibiting the activity of mitochondrial complex Ⅰ reduces the number of fibers of type Ⅰ [35]. The results were similar to those in our MSTN-deficient mice. Therefore, we believe that the change in energy metabolism after the MSTN gene knockout is one of the reasons for the change in the muscle fiber types. It is well known that different types of muscle fibers contain different amounts of mitochondria [36]. Type IIB muscle fibers contain fewer mitochondria [37]. In our results, the number of mitochondria per unit area in the myocytes of the *Mstn^−/−^* mice was lower than that of the wild-type mice. This also supports our speculation that the muscle fiber types may have changed and there may have been an increase in the type IIB fibers or a decrease in the type I fibers and type IIa fibers. However, the metabolic pathway of type IIB muscle fiber is mainly glycolysis rather than OXPHOS [38], so the change in the muscle fiber type also predicts the change in the muscle metabolism type in the *Mstn^−/−^* mice. A study has shown that an *Mstn* deficiency limits the shift toward oxidative metabolism during muscle activity [39]. In addition, ATP in the muscle of MSTN knockout mice decreased significantly after death [40]. The change in muscle fiber type is closely related to the change in energy metabolism. In addition to the MYH expression and cellular metabolism programs, the factors contributing to the fiber-type identities include multiple components of the sarcomere contractile machinery, such as fast and slow tropomyosin isoforms [41]. Therefore, we will continue to explore how MSTN affects muscle fiber types in future studies.

### 3.2. The Effect of Mstn^−/−^ on OXPHOS

The increase in muscle mass was not accompanied by a proportional increase in strength in the *Mstn^−/−^* mice, which has previously been speculated to be related to mitochondrial function [11]. Our study confirmed this; in the *Mstn^−/−^* mice, although the muscle fibers were thickened, the mitochondria in the muscle were thinner and smaller, the number per unit area was less, and the ATP production was significantly lower than in the wild-type mice. In muscle energy metabolism, mitochondria OXPHOS plays a prominent role in the cellular ATP generation dependent on respiratory chain complexes [42]. The previous study has shown that the genes related to ATP synthesis in the mitochondrial respiratory chain of MSTN propeptide transgenic mice are significantly down-regulated [19]. The MSTN loss down-regulates the expression of genes related to the mitochondrial respiratory chains complexes I, III, IV, and V, while the complexes II, Succinate dehydrogenase complex flavoprotein subunit A (SDHA), SDHB, and SDHC genes are significantly up-regulated [15]. Our results showed that the activity of the mitochondrial respiratory chain complexes I, III, IV, and V was indeed inhibited in the muscle of the *Mstn^−/−^* mice, and the expression of the glycolysis product pyruvate and key rate-limiting enzyme mRNA was significantly increased. This is consistent with previous studies. Apart from the above, we also found lower RMR and body temperature in the *Mstn^−/−^* mice, which is consistent with a reduction in mitochondrial complex activity.

### 3.3. The Effect of Mstn^−/−^ on Glycolysis

After the MSTN knockout, the activity of the respiratory chain complex is reduced, and the metabolism of OXPHOS in the muscle is switched to glycolytic metabolism [43]. A knockdown of MSTN up-regulates the expression of glucose transporters HK and PK and accelerates glycolysis [44]. It has also been shown that an MSTN deletion increases creatine kinase (CK) activity [45]. To mention CK, it is necessary to mention adenylate kinase (AK), which is involved in the energy conversion of ATP in muscles. AK is activated in anaerobic environments due to reduced mitochondrial synthesis [46]. The velocity of the AK can be increased 35-fold by oxygen deprivation in the intact rat diaphragm. The rate of increase in AK-catalyzed β-phosphate transfer coincided with the enhanced glycolytic flux [47]. In our study, if the glycolytic pathway is promoted, then adenylate kinase may be activated, and we will continue to investigate how adenylate kinase is powered in *Mstn^−/−^* mice in future studies. Previous studies showed that an MSTN knockout increased the activities of many enzymes involved in the glycolytic process [48] and accelerated glucose uptake and utilization in cattle [39,49]. We found elevated glucose levels in the muscle of the *Mstn^−/−^* mice, whereas previous studies showed decreased blood glucose in *Mstn^−/−^* mice [50]. Whether this involves the transport of glucose in muscle and blood remains to be investigated.

### 3.4. The Effect of Mstn^−/−^ on the TCA Cycle

The OXPHOS of NADH, but not FADH, contributed more than 60% of the ATP production [51]. In our study, the content of NADH in the muscle of the *Mstn^−/−^* mice was significantly reduced, while the content of NAD+ was increased. We believe that NADH acts as a substrate of complex I, and the reduction of its content limits the activity of complex I, thereby reducing ATP production [52,53]. NAD+ is a competitive substrate and inhibitor, which also limits the rate of NADH oxidation by complex Ⅰ, to some extent [54]. Therefore, we thought that the decrease of the NADH/NAD+ ratio in the *Mstn^−/−^* mice inhibited the production of ATP by OXPHOS. NADH is catalyzed by IDH, α-KGDH, and MDH [55]. There was no significant difference in the α-KGDH and MDH activities in the *Mstn^−/−^* mice (Figure 4d,e), and no significant difference in the *Kgdh* and *Mdh* gene expressions were observed between the *Mstn^−/−^* and WT groups (the data do not show). Therefore, IDH is worthy of attention. A study has shown that IDH2 and IDH3 are differentially expressed in different muscle fiber types. The type I slow fibers contained higher IDH2 and lower IDH3, while the opposite was true in type II fast fibers [56]. In the *Mstn^−/−^* quadriceps, the *Idh2* and *Idh3a* were up-regulated, which was associated with the fiber-type transition.

It is well known that NADH production is also mediated by fatty acid beta-oxidation. The mitochondrial β-oxidation of long-chain fatty acids is an important pathway for energy production in the skeletal muscle. Muscle CPT1 and CPT2 mediate fatty acid transfer to the mitochondrial matrix for β-oxidation [57]. SCAD is the initial rate-limiting enzyme for fatty acid β-oxidation, and the high expression of SCAD promotes the process of β-oxidation [57]. In our results, it was shown that all these genes were highly expressed. Studies have shown that MSTN knockout activates AMPK by up-regulating the AMP/ATP ratio, and the expression of PGC-1α as a target gene of AMPK is up-regulated. PGC-1α is involved in mitochondrial fatty acid uptake and oxidation [48]. PGC-1α can increase the β-oxidation of hepatocytes through Pparα [58]. This is consistent with the results of our study. This suggests that the decrease in NADH after the MSTN knockout is due to the inhibition of the TCA cycle.

### 3.5. Mstn^−/−^ Regulates Muscle Energy Metabolism through Smad2/3

We know that MSTN, as a member of the TGF-β superfamily, regulates the role of SMAD as a transcription factor by affecting the activation and expression of downstream signaling molecules [22]. It has been shown that TGF-β controls muscle size via Smad2/3 [59]. It is worth pondering whether Smad2/3 could regulate IDH in the TCA cycle. MSTN is known to regulate the activity of the transcription factor Smad2/3 through a cell membrane surface receptor. Smad3-null regenerated muscles decreased oxidative enzyme activity and impaired mitochondrial biogenesis [60]. In a study of MSTN knockout cattle, Smad2/3 bound to TET1 to regulate the DNA methylation modification to regulate the expression of myogenic factors to control muscle size [61], and it can also bind to the PDE5A promoter region to regulate glucose metabolism in the myocardium [25]. In *Mstn^−/−^* pigs, the reduction of Smad2/3 inhibited the atrophic effect of activin receptor-like kinase 5 (ALK5) [62]. Smad can bind to the promoter sequence of target genes and regulate gene transcription activity, thereby affecting gene expression. Given the above, we predict that the Smad binding site is located in the promoter sequences of *Idh2* and *Idh3a*. The ChIP-qPCR results confirmed our prediction and the gene expression of *Idh2* and *Idh3a* was suppressed due to the reduction of Smad2/3. Deletion of the *Idh2* decreased the TCA cycle intermediates NAD+, NADH, NADP+, and the NADPH decreased the number of mitochondrial cristae and changed the mitochondrial morphology [63], which is very similar to the *Mstn^−/−^* mice in this study. The cells transfected with *Idh2* resulted in increased ATP levels by increasing the OXPHOS, maintaining a higher intracellular ATP/AMP ratio, and improving the mitochondrial membrane potential, significantly increasing the cell baseline oxygen consumption [64]. Similarly, *Idh3a* is required for NADH to produce ATP in the mitochondrial respiratory chain [65], In a study of the retina, *Idh3a*-mutated mouse cell lines produced reduced levels of ATP and a reduced reserve capacity in the mitochondria of photoreceptor cells [66].

Generally, *Idh2* and *Idh3a* are important genes affecting mitochondrial function. Our results suggest that MSTN deletion leads to the downregulation of the downstream signaling molecule Smad2/3 of TGF-β, thereby inhibiting its role as a transcription factor and down-regulating the expression of IDH, a key rate-limiting enzyme in the TCA cycle. This also obstructs the process of the TCA cycle, resulting in the downregulation of the product NADH/NAD+ ratio, thus inhibiting the activity of OXPHOS and the production of ATP in the next step.

## 4. Materials and Methods

### 4.1. Ethics Statement

For the animal experiments, all the mice were grouped randomly, and the experimenters were blinded for group assignment and outcome assessment. All the experiments were carried out in strict accordance with the guidelines of the Experimental Animal Management and Operation Standards of Inner Mongolia University (IMU-MICE-2020-036).

### 4.2. Generation of Mstn^−/−^ Mice

Mice aged 6–8 weeks were selected as embryo donors and surrogates and housed in a conventional animal room with a temperature of (22 ± 1 °C), relative humidity of (55 ± 15%), a light/dark cycle of 12:12 h, and free access to food and water. Targeted mutations of exon 3 of *Mstn* with CRISPR/Cas9 system, as described previously [67], produced MSTN-deficient mice. The sgRNA sequence (TATAAGGCCAATTACTGCTCAGG) was cloned into the PCas9-Guide vector (GE100002, Origene, China). The constructs were injected into the fertilized eggs of B6D2F1 mice by pronucleus injection [68], and then the microinjected embryos were transferred into Kunming surrogate mice and produced the founders. Finally, the founders were mated with C57BL/6J mice, and the *Mstn* bi-allelic mutation (*Mstn^−/−^*) mice were identified by PCR and DNA sequencing. The generated *Mstn^−/−^* mice were fed under the same conditions as the WT mice after being separated into cages independently. After 8 weeks, the MSTN knockout mice and WT mice were killed by the cervical prolapse method, and fresh muscles were taken for energy metabolism-related detection. The PCR primers are shown in Supplementary Table S3. Non-editing C57BL/6J mice were used as the wild-type controls. The detailed preparation procedures for the *Mstn^−/−^* mice are described in previous literature [69].

### 4.3. Separation of Single Muscle Fibers

The mice were sacrificed by cervical prolapse and then sterilized with 75% ethanol. Their quadriceps muscles were removed at the correct site [30] in a sterile environment and placed in a preheated high-glucose DMEM medium. The quadriceps muscle bundles were washed 2 times using a high-glucose DMEM medium, and the type I collagenase digestion solution was digested in a 37 °C-water bath. The digestion state was observed until the muscle fibers were dispersed. The muscle bundle was gently blown using the small-size bore pipette to remove the external connective tissue until large amounts of muscle fibers were free. The termination medium, which was preheated to 37 °C, was added to horse serum-coated plates. The muscle fiber bundle was gently blown repeatedly with a small-size bore pipette. The intact single muscle fiber was washed in the DMEM medium with a fine-mouth elbow straw [70]. The quadriceps muscle fibers of the 6 WT and *Mstn^−/−^* mice were collected, the diameters of 3 muscle fibers were measured from each mouse, and the mean value was calculated. The diameters of 6 mice fibers were then compared with those of the control group.

### 4.4. RT-qPCR Assay

Total RNA was extracted respectively from the heart, muscle (quadriceps), liver, spleen, kidney, lung, pancreas, and brain tissues using an RNAiso Plus kit (9108, Takara, Kyoto, Japan). The RNA was then reversely transcribed to cDNA with a cDNA reverse transcription kit (RR820A, Takara, Kyoto, Japan). A real-time qPCR was then performed to detect the mRNA levels of specific genes and normalized to *Rpl7l1* [71] and α-tubulin (Supplementary Figure S1), as described previously [68]. Relative abundance was quantified using the 2^−ΔΔCt^ method. ΔΔCt = (Ct*Mstn*^−/−^ − Ct*Mstn*^−/−^-*Rpl7l1*) − (Ctwild type − Ctwild type-*Rpl7l1*). All the primer sequences were listed in Supplementary Table S4.

### 4.5. Western Blotting Analysis

The proteins were extracted from the quadriceps with a cell lysis buffer, separated by sodium dodecyl sulfate-polyacrylamide gel electrophoresis, and then electrically transferred to polyvinylidene fluoride membranes probed with antibodies specific for anti-MYH7 (1:1000, 22280-1-AP, ProteinTech Group, Chicago, IL, USA), anti-MYH2 (1:1000, 55069-1-AP, ProteinTech Group, Chicago, IL, USA), anti-MYH1 (1:1000, ab190605, Abcam, Cambridge, MA, USA), anti-MYH4 (1:1000, 20140-1-AP, ProteinTech Group, Chicago, IL, USA), anti-MSTN (1:1000, sc-134345, Santa Cruz Biotechnology, Santa Cruz, CA, USA), anti-Smad2+Smad3 (1:1000, ab202445, Abcam, Cambridge, USA), and anti-α-tubulin (1:2000, ab7291, Abcam, Cambridge, MA, USA), as previously described [68].

### 4.6. Transmission Electron Microscopy (TEM)

The TEM samples were prepared using the previous research methods [72]. Briefly, the quadriceps blocks were prepared and soaked immediately in 2.5% glutaraldehyde. After 6–8 h at 4 °C, they were cut into 1mm^3^ blocks. Next, the samples were rinsed with PBS (0.1 M) before being post-fixed by osmium tetroxide for 1–2 h. The muscle blocks were dehydrated through a graded series of alcohol and acetone. Subsequently, we used epoxy resin for embedding before slicing the ultra-thin sections. Then, double staining by uranyl acetate and lead citrate was performed. Finally, the images were acquired by a transmission electron microscope (JEM1400, JEOL, Tendo Japan) [72], and the images were collected by TEM image processing and analysis software (TIA). Image J software was used for the quantitative analysis. There were 6 mice in each group. TEM images of the quadriceps of each mouse were taken for 3 fields, and the length, width, and number of mitochondria were counted. After calculating the mean values of the 3 fields, the values of the 6 *Mstn^−/−^* mice were compared with those of the control group.

### 4.7. Mice Resting Metabolic Rate (RMR) and Body Temperature Assays

The oxygen consumption of the RMR was measured at 25 ± 0.5 °C with an animal respiratory metabolic measurement system (FMS, Sable System, Las Vegas, NV, USA). Each measurement period lasted 3 h, and the RMR was calculated as ml O_2_/g/h, as previously reported [73]. The body temperature was measured with a temperature probe implanted into the mouse’s abdominal cavity and recorded every 15 min using a DST nano-T (Star-Oddi, Gullbringusysla, Iceland) for 7 d.

### 4.8. Metabolic Substrates and Enzymes Assays

Different commercial kits for different compounds (COMIN Biotechnology, Suzhou, China) were used to assay their concentrations. The compounds included glucose (PT-1-Y), pyruvate (PA-1-Y), lactate (LA-1-Y), ATP (ATP-1-Y), and NADH/NAD+ (NAD-1-Y); the mitochondrial respiratory chain complexes I (FHTA-1-Y), III (FHTC-1-Y), IV (FHTD-1-Y) and V (FHTE-1-Y); and the key limiting enzymes in the TCA cycle of IDH (ICDHM-1-Y), α-KGDH (KGDH-1-Y), and MDH (MDHm-1-Y). The samples of 0.1 g fresh muscle tissues were respectively analyzed following the manufacturer’s instructions. Briefly, we put 0.1g tissue into a homogenizing tube containing 1ml extract from the kits, then put in about 15 ceramic beads, and homogenized low temperature with a homogenizer (Bertin, France). A Pierce BCA Protein Assay Kit (23227, Thermo Fisher, Waltham, MA, USA) was used to measure the protein concentration in the supernatant. The supernatant was collected and added to a 96-well plate, according to other reagents as instructed. After reading the absorbance values on a microplate spectrophotometer (Thermofisher, Waltham, MA, USA), the enzyme activity or metabolite concentration was calculated according to the formula, in which the protein concentration or weight (Supplementary Figure S2) was the normalization standard.

### 4.9. ChIP-qPCR

The ChIP was performed according to the Pierce Magnetic ChIP Kit (26157, Thermo Fisher, Waltham, MA, USA) guidelines. The chromatin was cross-linked and immunoprecipitated with 2 μg of anti-Smad2 + Smad3 (Abcam, Cambridge, USA, ab202445) and 30 μL of protein G beads overnight at 4 °C. The negative control was normal rabbit IgG. Finally, the purified immunoprecipitated chromatin was analyzed by a quantitative real-time PCR. According to previous research methods, the results of the ChIP-qPCR were normalized and presented as % input [74] The primer sequences are shown in Supplementary Table S5.

### 4.10. Statistical Analysis

All the data are expressed as the mean ± SD. In the graphs, all the bars represent the means, while each of the error bars represents one standard deviation. The statistical analyses were performed using the two-tailed unpaired Student’s *t*-test when comparing two groups with unequal standard deviations. * *p* < 0.05 and ** *p* < 0.01 *** *p* < 0.001 **** *p* < 0.0001 were considered statistically significantly.

## 5. Conclusions

A loss of MSTN function reduces ATP production by attenuating OXPHOS and inhibiting the TCA cycle, suggesting that a deficiency of MSTN disrupts the promotive regulation of Idh2 and Idh3a by the Smad2/3 in the TGF-β signaling pathway.

## Data Availability

The data generated and analyzed during this study are available upon reasonable request from the corresponding author.

## Supplementary Materials

The following supporting information can be downloaded at: https://www.mdpi.com/article/10.3390/ijms232415707/s1: Figure S1: The mRNA expression of genes involved in energy metabolism in the quadriceps of *Mstn^−/−^* and WT mice (the reference gene is α-tubulin); Figure S2: Enzyme activity and metabolite content related to energy metabolism in the quadriceps muscle of *Mstn^−/−^* and WT mice (normalized by weight); Table S1: The weight of different muscles from the wild-type and *Mstn*^−/−^ mice; Table S2: Organ and body weights from the wild-type and *Mstn*^−/−^ mice; Table S3: PCR primers used for the third exon of *Mstn* amplification; Table S4: Primers of Real-time qPCR; Table S5: Primers of CHIP-qPCR
