# Supplementary material for: Loss of Myostatin Alters Mitochondrial Oxidative Phosphorylation, TCA Cycle Activity, and ATP Production in Skeletal Muscle
Source: Int J Mol Sci. 2022 Dec 11;23(24):15707. doi: 10.3390/ijms232415707 (PMC9779574; doi:10.3390/ijms232415707)
Supplement: Supplementary file 1 [file ijms-23-15707-s001.zip › ijms-2017227-supplementary.pdf]

Supplementary Materials

Supplementary Figures

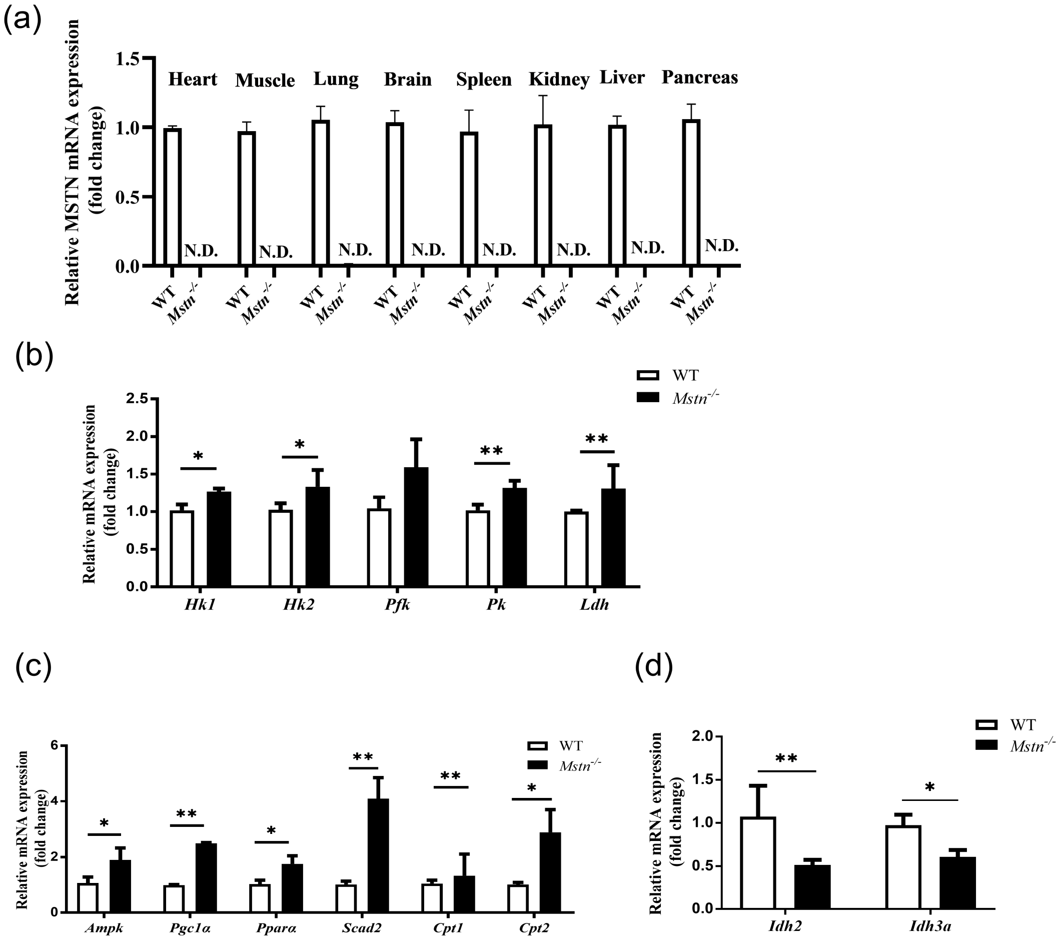

Figure S1. The mRNA expression of genes involved in energy metabolism in quadriceps of *Mstn*$^{-/-}$ and WT mice (the reference gene is α-tubulin). (a) Real-time PCR analyses of *Mstn* expression in different organs. N.D., not detectable. (b) Expression of mRNA for key rate-limiting enzymes involved in glycolysis in quadriceps. (c) Expression of mRNA for gene involved in β -oxidation in quadriceps. (d) Expression of mRNA for IDH. All data are presented as mean ± SD. Compared with the control group, $^{*}p< 0.05$, $^{**}p< 0.01$, Student's t-tests were used to calculate the *p*-values. We used n=6 mice per group. Except where noted, each dot presents a mouse.

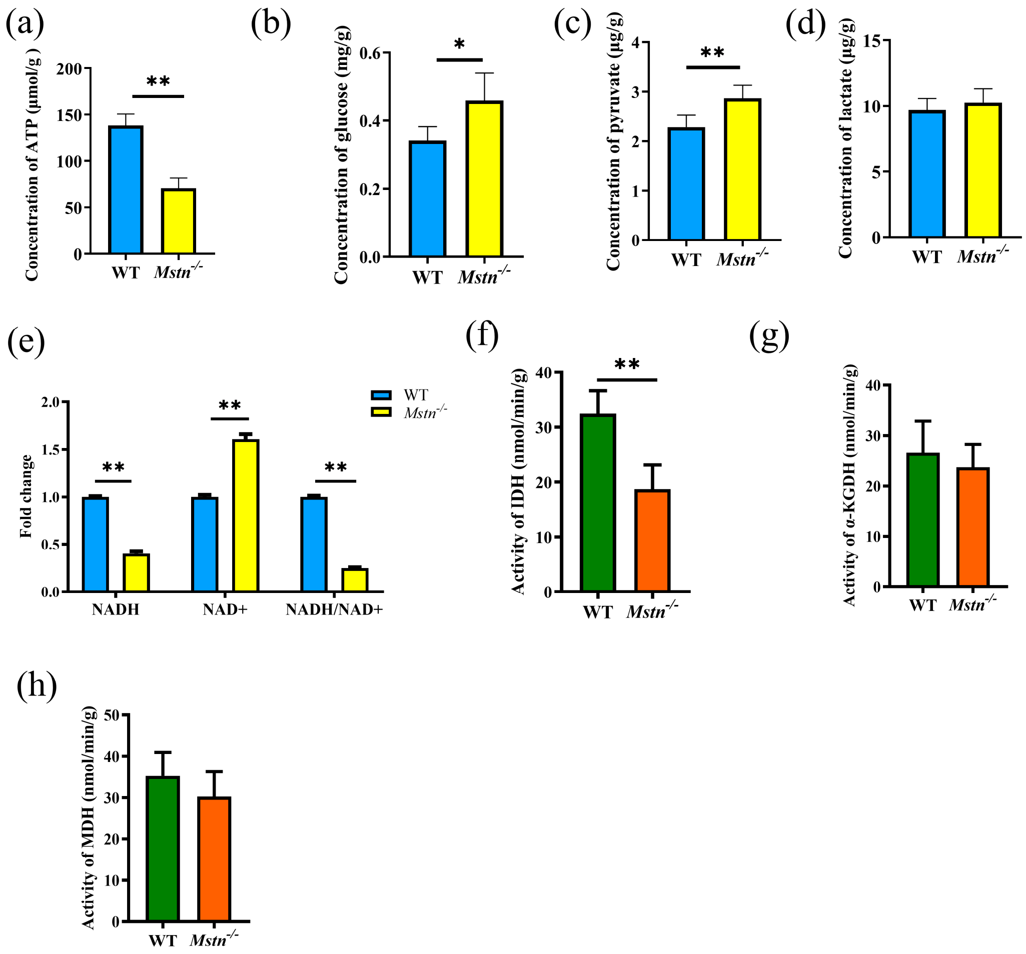

Figure S2. Enzyme activity and metabolite content related to energy metabolism in quadriceps muscle of *Mstn*$^{-/-}$ and WT mice (normalized by weight). (a-e) Concentrations of ATP, glucose, pyruvate, lactate, NADH, NAD+ in quadriceps of WT and *Mstn*$^{-/-}$ mice. (f-h) Activities of IDH, α -KGDH, MDH in quadriceps of WT and *Mstn*$^{-/-}$ mice. All data are presented as mean ± SD. Compared with the control group, **p*< 0.05, ***p*< 0.01, Student's t-tests were used to calculate the *p*-values. We used n=6 mice per group. Except where noted, each dot presents a mouse.

Supplementary Tables

Table S1. The weight of different muscles from wild-type and *Mstn*$^{-/-}$ mice

Weight (mg)	Soleus	Plantaris	Gastrocnemius	Tibialis anterior	Quadriceps
WT（♂）	6.13±0.33	13.59±0.79	139.89±5.22	60.95±5.44	219.41±9.14
Mstn$^{-/-}$（♂）	6.86±0.66*	15.63±1.52*	155.75±6.81**	77.56±8.4**	261.77±16.43***
WT（♀）	5.23±0.28	12.01±0.75	124.31±6.27	53.65±4.73	195.63±15.32

Mstn$^{-/-}$（♀）	5.74±0.39*	12.77±0.57	133.41±2.81**	62.51±7.62*	218.46±9.25*

N=6 mice per group; 8 weeks old; Different from control, t-test *$p < 0.05$, **$p < 0.01$, ***$p < 0.001$

Table S2. Organ and body weights from wild-type and *Mstn*$^{-/-}$ mice

Weight (g)	Body	Heart	Kidney	Liver	Spleen	Lung	Brain	Ovary	Testis
WT（♂）	27.16±0.57	0.2±0.02	0.41±0.03	1.23±0.12	0.10±0.02	0.20±0.01	0.41±0.02	—	0.17±0.03
Mstn$^{-/-}$（♂）	31.29±1.06*	0.25±0.02*	0.48±0.05*	1.43±0.06*	0.08±0.03	0.21±0.02	0.39±0.01	—	0.15±0.01
WT（♀）	25.99±0.89	0.16±0.03	0.42±0.02	1.38±0.14	0.10±0.01	0.21±0.01	0.42±0.02	0.01±0.00	—
Mstn$^{-/-}$（♀）	29.54±3.29*	0.21±0.01*	0.45±0.02*	1.72±0.20*	0.10±0.05	0.20±0.1	0.42±0.02	0.01±0.01	—

N=6 mice per group; 8 weeks old; *Different from control, t-test $p < 0.05$

Table S3 PCR primers used for the third exon of *Mstn* amplification

**Gene**	**Forward Primer (5'-3')**	**Reverse Primer (5'-3')**
Mstn 3ex	AGTCAAGGTGACAGACACACCC	GTGCTTGAATTCACAGTTTCGA

Table S4 Primers of Real-time qPCR

**Gene**	**Forward Primer (5'-3')**	**Reverse Primer (5'-3')**
Rpl7l1	ACGGTGGAGCCTTATGTGAC	TCCGTCAGAGGGACTGTCTT
α-tubulin	TGTCCTGGACAGGATTCGC	CTCCATCAGCAGGGAGGTG
Mstn	AGTGGATCTAAATGAGGGCAGT	GTTTCCAGGCGCAGCTTAC
Myh7	AGTCCCAGGTCAACAAGCTG	TTCCACCTAAAGGGCTGTTG
Myh2	AGTCCCAGGTCAACAAGCTG	GCATGACCAAAGGTTTCACA
Myh1	AGTCCCAGGTCAACAAGCTG	CACATTTTGCTCATCTCTTTG
Myh4	AGTCCCAGGTCAACAAGCTG	TTTCTCCTGTCACCTCTCAACA
Hk1	CAAGAAATTACCCGTGGGATTCA	CAATGTTAGCGTCATAGTCCCC
Hk2	CTAAGGGGTTCAAGTCCAGTGG	AGACCAATCTCGCAGTTCTGA
Pfk1	GCGACTTGCTGAATGATCTCC	CATTGTCGATTGAGCCAACCA
Pk	GAACATTGCACGACTCAACTTC	CAGTGCGTATCTCGGGACC
Ldh	CAAAGACTACTGTGTAACTGCGA	TGGACTGTACTTGACAATGTTGG

Scad	TTGCCGAGAAGGAGTTGGTC	AGGTAATCCAAGCCTGCACC
Cpt1	TGTCTACCTCCGAAGCAGGA	GCCATGACCGGCTTGATCT
Cpt2	TGACAGCCAGTTCAGGAAGAC	GGCCTGAGATGTAGCTGGTG
Ampk	GTCATGATAGCTTGCATAAATGGTG	AGTTGAATAGAACAAGCCCTGGAC
Pgc1α	CATTTGATGCACTGACAGATGGA	CCGTCAGGCATGGAG GAA
Pparα	GGCAAAAGGCAAGGAGAAGCA	GGGAAAGAGGTGAGAGAAGAGC

Table S5 Primers of CHIP-qPCR

**Gene**	**Forward Primer (5'-3')**	**Reverse Primer (5'-3')**
Idh2	GTGTCACTGGGTGGAGGT	TTACTTAGGGCTGGCTTAC
Idh3a	AGTGCTTCATGGCTTCAT	GGTTAGGTGCTTGTTTCA
